# Sub-threshold autism traits: The role of trait emotional intelligence and cognitive flexibility

**DOI:** 10.1111/bjop.12033

**Published:** 2013-05-15

**Authors:** Elif Gökçen, Konstantinos V Petrides, Kristelle Hudry, Norah Frederickson, Luke D Smillie

**Affiliations:** 1 London Psychometric Laboratory, University College LondonUK; 2 Olga Tennison Autism Research Centre, School of Psychological Science, La Trobe UniversityAustralia; 3 Research Department of Clinical, Educational and Health Psychology, University College LondonUK; 4 Melbourne School of Psychological Sciences, The University of MelbourneAustralia

## Abstract

Theory and research suggests that features of autism are not restricted to individuals diagnosed with autism spectrum disorders (ASDs), and that autism-like traits vary throughout the general population at lower severities. The present research first investigated the relationship of autism traits with trait emotional intelligence and empathy in a sample of 163 adults aged between 18 and 51 years (44% male). It then examined performance on a set of tasks assessing social cognition and cognitive flexibility in 69 participants with either high or low scores on ASD traits. Results confirm that there is pronounced variation within the general population relating to ASD traits, which reflect similar (though less severe) social-cognitive and emotional features to those observed in ASDs.

Social cognition is a multi-dimensional concept comprising interrelated operations that facilitate effective social functioning. Such operations include the processing of emotional information, perception of social cues, and ability to mentalize and to make judgements about social relationships (Adolphs, 2003). Deficits in social cognition are particularly salient in individuals diagnosed with autism spectrum disorders (ASDs), which manifest severe impairments in processes such as emotion perception, face processing, mentalizing and empathic understanding (Ashwin, Chapman, Colle, & Baron-Cohen, 2006; Baron-Cohen & Wheelwright, 2004; Baron-Cohen, Wheelwright, Hill, Raste, & Plumb, 2001; Frith & Frith, 2003).

Converging research suggests that all individuals vary along a dimension of social-cognitive ability, ranging from typical development, through ASD, and with classic autism at the extreme end (Baron-Cohen, Wheelwright, Skinner, Martin, & Clubley, 2001; Constantino, 2011). In support of this continuous view, it has been proposed that undiagnosed relatives of individuals with ASDs may have a genetic disposition towards the expression of the broader autism phenotype, a set of milder, but qualitatively similar, traits relating to social, cognitive, and language difficulties (Bishop *et al*., 2004; Piven *et al*., 1997). In addition, there is growing evidence that the expression of sub-threshold ASD traits may go beyond relatives of those diagnosed with an ASD and extend into the general population (Baron-Cohen, Wheelwright, Skinner, *et al*., 2001; Constantino, 2011; Hoekstra, Bartels, Cath, & Boomsma, 2008; Jones, Scullin, & Meissner, 2011).

The continuum approach has led to the development of the Autism Spectrum Quotient (AQ; Baron-Cohen, Wheelwright, Skinner, *et al*., 2001), an empirically based self-report measure that aims to quantitatively assess features related to the impairments characteristic of ASDs. To date, numerous studies have produced evidence supporting the AQ as a valid measure of the broader autism phenotype (Baron-Cohen, Wheelwright, Skinner, *et al*., 2001; Hoekstra *et al*., 2008; Jones *et al*., 2011; Russell-Smith, Maybery, & Bayliss, 2011). For instance, some studies have found that unaffected parents of children with ASDs scored higher than control parents on the AQ (Bishop *et al*., 2004; Wheelwright, Auyeung, Allison, & Baron-Cohen, 2010). Further, Jobe and White (2007) reported that higher scores on the AQ were associated with increased interpersonal difficulties in typically developing young adults.

The AQ measure may be related to non-clinical social-cognitive constructs studied in basic personality research, such as trait emotional intelligence (trait EI, also known as trait emotional self-efficacy; Petrides, 2009). Trait EI is defined as a constellation of emotional self-perceptions located at the lower levels of personality hierarchies (Petrides, Pita, & Kokkinaki, 2007). Many aspects of social and emotional functioning that appear to be impaired in ASD (i.e., adaptability, empathy, social awareness, and communication) are encompassed by trait EI (Petrides, Hudry, Michalaria, Swami, & Sevdalis, 2011). In addition, research has documented atypical trait EI profiles among individuals with ASDs (Montgomery *et al*., 2008; Petrides *et al*., 2011), and has further demonstrated that trait EI can potentially provide important insights into the underpinnings of optimal social-cognitive functioning (e.g., the flexible application of complex emotions and social cue decoding). This suggests meaningful overlap between the construct of trait EI and our understanding of impairments in ASDs.

Some researchers propose that deficits in executive functions, such as cognitive flexibility, may account for the social-cognitive impairments associated with ASDs (Ozonoff, 1997; Schopler, Mesibov, & Kunce, 1998). Cognitive flexibility concerns the extent to which one is able to disengage attention from a target and shift to a different thought or action, in response to changing environmental demands (Canas, Quesada, Antoli, & Fajardo, 2003). Failure to respond flexibly and adaptively to novel situations or changing environments manifests in persistent and rigid behaviour. This is readily observed in ASDs, which are characterized by narrow interests, poor attention-switching, difficulties in assimilating to change or novelty, and engagement in repetitive behavioural patterns (Baron-Cohen, 2008; Hill, 2004). Interestingly, impairments in such processes are not only postulated as the root of repetitive and inflexible behaviours that define ASDs, but are also suggested to underlie the core deficits in social cognition (Yoshida *et al*., 2010). This may be due to the complex interplay between executive function and emotion. For example, it has been argued that emotional states enhance the flexibility with which information is processed and interpreted (Ashby, Isen, & Turken, 1999). Conversely, impaired cognitive flexibility may reduce one’s ability to effectively attend to, process, and use social and emotional information.

Literature has suggested that the ability to accurately process socioemotional cues from a stream of complex (whether verbal, non-verbal, or contextual) information is primarily dependent upon executive functions, such as cognitive flexibility (Bull, Phillips, & Conway, 2008). However, the nature of this relationship remains unclear in ASDs and is relatively unexplored in relation to sub-threshold ASD traits found in the general population. It is important to understand how these processes are related and the most suitable approach to assess this may be via tasks that provide the opportunity to test the domains of executive functioning and social cognition simultaneously.

In the present research, we first assessed the association of sub-threshold ASD traits with trait EI and empathy (Study 1), and subsequently examined performance on tasks measuring social cognition and cognitive flexibility separately and simultaneously in a sub-group of participants with either high or low ASD traits (Study 2).

Based on the reviewed literature, it was hypothesized that:

1. AQ scores would be negatively associated with global trait EI, and its consistent factors of Well-being, Self-control, Emotionality, and Sociability (H1) and negatively correlated with empathy scores (H2). Conversely, trait EI was predicted to be positively associated with empathy scores (H3).
2. A positive association was expected between social-cognitive ability and cognitive flexibility (H4). Furthermore, compared to their peers with lower ASD traits, individuals with higher ASD traits were expected to demonstrate poorer performance on tasks measuring social cognition and cognitive flexibility (H5). It was further predicted that high ASD traits would be associated with poorer performance on the emotional rule-shift test (ERST); a newly developed task that incorporates emotionally relevant stimuli into a well-established set-shifting paradigm (H6).

## Study 1

### Method

#### Participants

Participants were an opportunity sample of 163 adults (44% male) aged between 18 and 51 years (*M* = 23.49, *SD* = 5.16), recruited via emails advertising the study and an online human subject pool. Our sample consisted of London-based university students, university graduates, and volunteers from the local area.

#### Measures

##### ASD traits

The AQ (Baron-Cohen, Wheelwright, Skinner, *et al*., 2001) is a 50-item questionnaire measuring ASD traits in adults of normal intellectual ability. It comprises five dimensions corresponding to the social-cognitive deficits associated with ASD: social skills, attention-switching, attention to detail, communication, and imagination. The AQ requires individuals to indicate whether they ‘strongly agree’, ‘slightly agree’, ‘slightly disagree’, or ‘strongly disagree’ with each item, half of which are worded to elicit an ‘agree’ response and the other half, a ‘disagree’ response in participants. Individuals score in the range of 0–50, with higher scores reflecting more severe symptomatology. The AQ has been shown to distinguish between groups of individuals with ASD and age-matched controls (Baron-Cohen, Wheelwright, Skinner, *et al*., 2001).

##### Trait emotional intelligence

Trait EI was profiled using the TEIQue (Petrides, 2009), a 153-item inventory that provides comprehensive assessment of the trait EI sampling domain. This measure yields scores on 15 emotion-related facets, four factors and global trait EI (see Table 2003). Participants are required to respond on a 7-point Likert scale ranging from *completely disagree* to *completely agree*. The TEIQue has been shown to have sound predictive validity and sound psychometric properties more generally (Gardner & Qualter, 2010; Martins, Ramalho, & Morin, 2010; Mikolajczak & Luminet, 2008; Mikolajczak, Luminet, Leroy, & Roy, 2007).

**Table 1:** The sampling domain of trait emotional intelligence (EI) in adults

High scorers perceive themselves as…	
Well-being	
Self-esteem	…successful and self-confident.
Trait happiness	…cheerful and satisfied with their lives.
Trait optimism	…confident and likely to ‘look on the bright side’ of life.
Self-control	
Emotion control	…capable of controlling their emotions.
Stress management	…capable of withstanding pressure and regulating stress.
Impulsiveness (low)	…reflective and less likely to give into their urges.
Emotionality	
Emotion perception (self and others)	…clear about their own and other people’s feelings.
Emotion expression	…capable of communicating their feelings to others.
Relationships	…capable of having fulfilling personal relationships.
Trait empathy	…capable of taking someone else’s perspective.
Sociability	
Social awareness	…capable of taking someone else’s perspective.
Emotion management (others)	…capable of influencing other people’s feelings.
Assertiveness	…forthright, frank, and willing to stand up for their rights.
Independent facets	
Adaptability	…flexible and willing to adapt to new conditions.
Self-motivation	…driven and unlikely to give up in the face of adversity.

*Note*. The two ‘independent facets’ feed directly into global trait EI without going through any factor.

##### Empathy

The Empathy Quotient (EQ) is a 40-item questionnaire measuring global empathy (Baron-Cohen & Wheelwright, 2004), in both typically developing individuals and clinical populations with empathic dysfunction (e.g., ASDs). Each item presents a statement regarding preferences and habits, with responses made on a 4 point scale ranging from *strongly disagree* to *strongly agre*e. Individuals score in the range of 0–80, with higher scores reflecting greater empathy. Like the AQ, this measure has also been shown to differentiate between clinical and control groups, with significantly lower EQ scores reported for groups of individuals with ASDs.

### Results

The means, standard deviations, internal consistency reliability estimates, and inter-correlation coefficients for all variables can be seen in Table 1999. As hypothesized, global trait EI, and the TEIQue factors of Well-being, Emotionality, and Sociability were all negatively correlated with AQ scores, and positively correlated with EQ scores. While analysis revealed a positive correlation between the TEIQue factor of Self-control and EQ, the negative correlation with AQ did not reach statistical significance.

**Table 2:** Descriptive statistics, internal consistencies and correlations among the Autism Spectrum Quotient, factors of the Trait Emotional Intelligence Questionnaire, and Empathy Quotient

	*M*	*SD*	α	1	2	3	4	5	6
1. AQ	17.41	6.89	.80						
2. TEIQue Global Trait EI	4.54	.61	.91	−.39**					
3. TEIQue Well-being	4.80	.92	.86	−.31**	.90**				
4. TEIQue Self-control	4.27	.66	.73	−.12	.61**	.47**			
5. TEIQue Emotionality	4.65	.83	.84	−.41**	.87**	.73**	.28**		
6. TEIQue Sociability	4.47	.69	.80	−.34**	.81**	.67**	.30**	.69**	
7. EQ	43.01	13.72	.91	−.62**	.50**	.42**	.19*	.58**	.32**

*Note*. *N* = 163.

AQ = Autism Spectrum Quotient; TEIQue = Trait Emotional Intelligence Questionnaire; EQ = Empathy Quotient.

*p < .05; **p < .01, two tailed.

The relationship between ASD traits and trait EI was assessed in greater detail through forced-entry regression analyses.^1^ The total AQ score was regressed onto the trait EI factors of Well-being, Self-control, Emotionality, and Sociability. The regression model was significant, *R* = .42, *F*(4, 158) = 8.40, *p* < .001, and the four predictors together explained 18% of the variance in AQ scores. Emotionality, β = −.35, *t*(158) = 3.05, *p* = .003, emerged as a significant predictor of ASD traits in the equation. The sign of the coefficient suggests that low Emotionality scores were related to higher levels of ASD traits. None of the other predictors reached statistical significance, Sociability, β = −.012, *t*(158) = 1.09, *ns*, Well-being, β = .03, *t* < 1, *ns*, and Self-control, β = −.003, *t* < 1, *ns*. Then, to establish the shared variance among the four predictors, we subtracted the sum of all squared semi-partial correlations^2^ from the proportion of variance explained by the four predictors, *R*²(.18) – total *sr*^2^(.06) = .12. This finding demonstrates that there is substantial shared variance among the four trait EI factors which overlaps with variance in AQ scores.

Next, the total AQ score was regressed onto the three facets of trait EI most strongly correlated with ASD traits.^3^ Once again, the regression model was significant, *R* = .53, *F*(3, 159) = 20.61, *p* < .001, and together, the three predictors explained 28% of the variance in AQ scores. Social awareness, β = −.29, *t*(159) = 3.52, *p* = .001, and adaptability, β = −.23, *t*(159) = 3.13, *p* = .001, were both significant predictors of ASD traits. The signs of the coefficients indicate that lower social awareness and adaptability were linked to higher levels of ASD traits. Empathy, β = −.15, *t*(159) = 1.80*, p* = .074, however, did not emerge as a significant predictor in the model. Finally, to establish the shared variance among the three predictors, we subtracted the sum of all squared semi-partial correlations^4^ from the proportion of variance explained by the three predictors, *R²*(.28) – total *sr*^2^(.11) = .17. Again, this finding demonstrates that substantial shared variance among the three trait EI facets is driving the variance in AQ scores. Taken together, these results provide partial support for H1 and full support for H2. In addition, the negative correlation between AQ and EQ scores provide support for H3.

## Study 2

### Method

#### Participants

A subset of participants from Study 1 – identified as ‘High AQ’ or ‘Low AQ’ according to cut-offs on the AQ questionnaire (see Baron-Cohen, Wheelwright, Skinner, *et al*., 2001) – were invited to take part in Study 2. Participants with a score over 20 on the AQ were identified as ‘High AQ’, whilst participants with a score of 13 and below were identified as ‘Low AQ’. Those meeting the criteria for Study 2 were invited to schedule an appointment with the researcher, whilst those with scores in the mid-range were thanked for their time and sent a copy of the study debrief.

Seventy-one of the 79 individuals identified as ‘High AQ’ and ‘Low AQ’ agreed to take part in Study 2. Data from two of these participants were excluded for the following reasons: one for showing very low accuracy on the EYES test (3.2 SDs below the group mean in the ‘High AQ’ group), and the other due to an error in ERST administration (‘Low AQ’ group). Of the remaining sample, there were 35 participants aged between 19 and 46 (*M* = 25.48, *SD* = 7.22, 18 males) in the ‘High AQ’ group, and 34 participants aged between 20 and 32 (*M* = 23.21, *SD* = 3.31, 14 males), in the ‘Low AQ’ group. Chi-square analysis revealed no significant differences in gender distribution between the high AQ and Low AQ groups, χ^2^(1) = 0.729, *p* = .393. All participants were either current university students or university graduates. Additional information for the high and low ASD groups is included in Table 2006.

**Table 3:** Descriptive statistics for the key variables in high and low autism spectrum disorder trait groups

	Low AQ (*n* = 34)	High AQ (*n* = 35)
Age		
*M*	22.85	23.51
*SD*	3.40	6.11
Range	18–32	18–46
Total AQ Score		
*M*	10.21	24.97
*SD*	2.28	3.00
Range	4–13	21–32
TEIQue global trait EI		
*M*	4.96	4.31
*SD*	.46	.42
Range	3.95–6.11	3.53–5.18
TEIQue Well-being		
*M*	5.30	4.61
*SD*	.81	.82
Range	3.65–6.70	2.66–6.03
TEIQue self- control		
*M*	4.35	4.12
*SD*	.61	.60
Range	3.31–6.08	2.68–5.16
TEIQue Emotionality		
*M*	5.29	4.37
*SD*	.56	.53
Range	4.18–6.46	3.31–5.57
TEIQue Sociability		
*M*	5.02	4.21
*SD*	.62	.50
Range	3.85–6.49	2.73–5.23
EQ		
*M*	48.68	35.26
*SD*	10.75	11.66
Range	24–72	15–61

*Note*. AQ = Autism Spectrum Quotient; TEIQue = Trait Emotional Intelligence Questionnaire; EQ = Empathy Quotient.

#### Measures and procedure

##### Social cognition

The revised Reading the Mind in the Eyes Test (EYES; Baron-Cohen, Wheelwright, Hill, *et al*., 2001) is an advanced social cognition task assessing the ability to decode the mental state of an individual based on 36 images of the eye region of the face. Using only the visual information, respondents are required to select one of four forced-choice mental state descriptors (one target word and three foil words of the same emotional valence) that best portrays the thoughts or feelings expressed by the pictured individuals. The test stimuli contain complex mental states only (e.g., ‘pensive’ and ‘elated’, rather than simple states like ‘happy’ or ‘sad’), which constitutes an important methodological innovation in the literature. Participants were also provided with a glossary defining all the mental state terms used in the task, and were asked to read and indicate any meanings of which they were unsure.

##### Cognitive flexibility

Participants completed a computerized set-shifting task (Smillie, Cooper, Tharp, & Pelling, 2009) based upon the Wisconsin Card Sorting Test (WCST; Grant & Berg, 1948), programmed in Matlab using the Psychophysics Toolbox extensions (Brainard, 1997; Pelli, 1997). Each trial consisted of the presentation of a single card, which varied in three different ways: (a) was blue or yellow in colour, (b) displayed either a ‘0’ or an ‘X’ on the front, and (c) appeared on the left or right side of the screen. Participants were instructed to categorize cards into two piles based on one of the three sorting rules by pressing either the ‘\’ or ‘/’ key (which were labelled as ‘A’ and ‘B’), upon the presentation of each stimulus. After each trial, participants were provided with feedback indicating whether the response made was *correct!* or *incorrect!*.

Participants were told that to learn how to sort the cards accurately, they would need to use the feedback and learn by trial-and-error. An unannounced shift in the sorting rule occurred after the participant had made 10 consecutive correct responses. In total, five shifts took place during the experiment with each of the rules repeating twice. The task duration was approximately 10 min and finished once the participant had successfully completed all five shifts or when the maximum number of trials (120), had been reached. Performance was assessed in terms of the *shifting efficiency* measure proposed by Cianchetti, Corona, Foscoliano, Scalas, and Sannio-Fancello, (2005). According to this scoring method, a participant is awarded six points for each shift that is successfully completed and an additional point for each remaining trial, provided all five shifts are made before reaching 120 trials. For instance, a participant who has made all five shifts in 100 trials would receive a shifting efficiency score of 5*6 + (120–100) = 50.

##### Social cognition and cognitive flexibility

The ERST seeks to index perseverative response styles in a social context by incorporating emotionally relevant stimuli in a cognitive flexibility task based directly upon the WCST. This task has some similarities with other assessments of emotion-based cognitive control (e.g., Affective Shift Task; De Lissnyder, Koster, Derakshan, & De Raedt, 2010).

The ERST incorporates a standardized and open source battery of close-up pictures of faces (NimStim, http://www.macbrain.org/resources.htm; Tottenham, Tanaka, Leon, McCarry, & Nurse, 2009). A total of 22 colour images of male (11) and female (11) faces were selected from this battery. Each trial consisted of the presentation of a single card depicting a close-up image of a face varying on (a) valence (positive vs. negative emotion) and (b) expressiveness/activation (strong vs. weak). The ERST comprised images of 10 positively and 12 negatively valenced facial expressions. Each image was dual-dimensional and differed in two ways (e.g., strong-positive, strong-negative, weak-positive, weak-negative), capturing weak and strong expressions of basic emotions (e.g., happiness, sadness, anger, fear, and disgust). According to Barrett and Russell (1999), the structure of affect can be captured through the two dimensions of valence and activation.

The ERST requires participants to respond to stimuli by sorting cards into two teams (team A vs. team B), based on either valence or expressiveness. Sorting categories were given social labels such as ‘team A’, rather than ‘stack A’, due to the use of facial stimuli. There were a total of 44 trials (2 decks of 22 cards) and two rule shifts. Participants were told that they must sort the card into two categories by assigning them to either ‘Team A’ or ‘Team B’ and received feedback on accuracy (*correct* or *incorrect*) at the end of each trial. Participants were further informed that they would have to rely on the feedback to learn by trial-and-error how to sort out the cards correctly.

The rule for sorting the cards was initially based on valence, where the participant would learn to assign positive expressions to ‘team A’ and negative expressions to ‘team B’. After 10 consecutive correct responses were made, an unannounced rule-shift for card sorting was implemented and the valence-based rule shifted to the expressiveness-based rule. In total, two rule-shifts took place during the experiment with the rule sequence always following the order of valence-expression-valence. The task terminated once the participant had successfully completed both shifts, or when a maximum of 44 trials had been reached. Similar to the WCST, performance on the ERST was assessed based on shifting efficiency (how successfully the respondent shifted from the first to the second rule and back again). Cianchetti and colleagues’ proposed scoring method was used in this case too. Thus, a participant was awarded six points for each shift that had been successfully completed and an additional point for each remaining trial provided both shifts were made before reaching the maximum number of trials.

### Results

First, we performed a bivariate correlation analysis to test the association between social cognition and cognitive flexibility (H4). As expected, results revealed a significant positive relationship between EYES and WCST scores, *r* = .33, *p* = .005.

**Figure 1:**
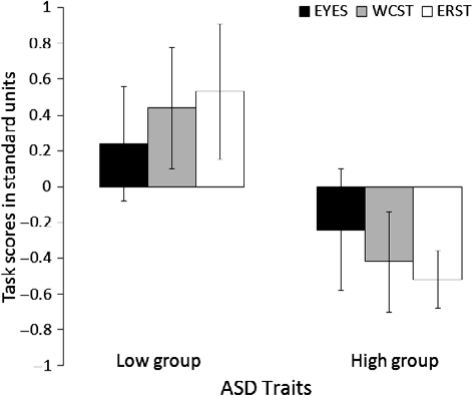
Individuals with lower ASD traits (and high trait emotional intelligence [EI]) demonstrate better emotion perception on the EYES test and display greater set-shifting efficiency on the Wisconsin Card Sorting Test and Emotional Rule-Shift Test, compared to individuals with higher ASD traits (and low trait EI). Error bars represent 95% confidence interval. Graph is based on standardized dependent variables.

Next, we examined group differences in task performance using multivariate analysis of variance (ANOVA). Total scores on the EYES, WCST, and ERST tasks were entered as dependent variables and participant group (high AQ vs. low AQ group), as the independent variable. The effect of group was highly significant, *F*(3, 65) = 15.75, *p* < .001, and explained over 40% of the variance in the composite variable, η_p_^2^ = 0.42 (see Figure 1).

Inspection of follow up ANOVAs revealed a significant effect of group on the EYES test *F*(1, 67) = 4.14, *p* = .046, η_p_^2^ = 0.06, with the low AQ group (*M* = 28.26, *SD* = 4.71) performing better than the high AQ group (*M* = 25.83, *SD* = 5.22). Furthermore, there was a significant main effect of group on shifting efficiency on the WCST, *F*(1, 67) = 15.51, *p* < .001, η_p_^2^ = 0.19, with low AQ participants (*M* = 26.62, *SD* = 16.15) demonstrating superior performance in comparison to high AQ participants (*M* = 12.63, *SD* = 13.26). Finally, analysis returned a significant main effect of group on shifting efficiency on the ERST, *F*(1, 67) = 26.30, *p* < .001, η_p_^2^ = 0.28, with low AQ participants (*M* = 4.29, *SD* = 4.00) performing better than high AQ participants, who performed at near floor level (*M* = .51 *SD* = 1.70). Overall, these findings yield strong support for the hypothesis that individuals with higher levels of ASD traits would exhibit poorer performance on tasks measuring social cognition, cognitive flexibility, and emotional set-shifting (H5 and H6).

### Discussion

The aim of this study was twofold: first, to assess the relationship between ASD traits and trait EI, and second, to examine performance on tasks measuring social cognition and cognitive flexibility in non-diagnosed participants with extreme ASD trait scores.

The AQ scores were negatively correlated with global trait EI, the TEIQue factors of Well-being, Emotionality, and Sociability, and Empathy. As expected, analysis also revealed a positive correlation between trait EI and empathic functioning. Collectively, these findings demonstrate a strong overlap among measures of empathy, trait EI and ASD traits. However, in contrast to the positive association between Self-control and EQ, the negative association between this TEIQue factor and AQ scores did not reach statistical significance. This result is in line with findings from clinical samples reporting similar levels of self-control among individuals with AS and typically developing controls (Konig & Magill-Evans, 2001). In addition, the high negative correlation between AQ and EQ scores replicates findings reported in previous studies (Wheelwright *et al*., 2006; Wright & Skagerberg, 2012).

Further analysis revealed Emotionality as the only TEIQue factor to reach statistical significance, suggesting that individuals with higher levels of ASD traits have difficulties in expressing their emotions and taking another person’s perspective. Analysis also revealed two TEIQue facets as incremental predictors of ASD trait scores. Adaptability and social awareness were both negatively associated with AQ scores, suggesting that participants with higher sub-clinical ASD traits experience difficulties with flexible behaviour and interpersonal competency. This is an important finding indicating that impairments in trait EI facets central to effective socioemotional functioning exist at the more limited expression of ASD traits, as well as in clinical populations (Petrides *et al*., 2011). Overall, these results suggest that individuals with higher levels of ASD traits have lower trait EI and empathy (see also Petrides *et al*., 2011).

Our findings also revealed a positive association between social cognition and cognitive flexibility. Given that the high ASD trait group showed poor performance on both the EYES and WCST, this result suggests that there may be a shared factor underpinning difficulties in social cognitive functioning and cognitive flexibility.

In addition, the group with higher ASD traits demonstrated significantly poorer performance on the EYES test than the group with lower scores. As in previous studies (e.g., Baron-Cohen, Wheelwright, Skinner, *et al*., 2001), high scores on the AQ were linked to deficits in decoding internal mental states based on nonverbal cues. The prediction that those with higher ASD trait scores would perform more poorly on the WCST relative to those with lower ASD trait scores was supported. Participants with high ASD traits had poorer shifting efficiency and more perseverative errors, thus yielding further support for the association between supervisory processes, such as cognitive flexibility, and social-cognitive abilities (Fisher & Happe, 2005; Ozonoff, 1997; Pellicano, 2007).

Those with higher ASD scores showed far poorer ERST performance than their low trait peers. As on the WCST, poorer shifting efficiency and a higher incidence of perseverative errors were observed among participants with higher ASD trait scores when manually categorizing emotion-related stimuli. The fact that the high AQ group performed at near floor level, while the low AQ group performed well, suggests that the flexible processing of emotional stimuli demanded by this task may have captured a critical difference between high and low AQ scorers. It further suggests that combining cognitive flexibility with social-cognitive functioning leads to an additive effect on difficulty level for individuals with higher ASD trait scores. Taken together, our findings yield strong support for the hypothesis that individuals with sub-threshold ASD traits may experience qualitatively similar (though less severe) difficulties in cognitive flexibility as individuals diagnosed with ASD. To our knowledge, this is the first study to assess the flexible processing of social-emotional information in relation to sub-threshold autism traits.

As noted earlier, past research has shown lower trait EI and empathy profiles in individuals with ASDs (Baron-Cohen & Wheelwright, 2004; Petrides *et al*., 2011). Consistent with these findings, this study reported significantly lower trait EI and empathy scores for high versus low ASD trait participants. This finding is of particular importance because none of the participants reported an existing diagnosis of ASD, and all individuals scored at or below the suggested clinical cut-off point (32+) for ASD traits as measured by the AQ (Baron-Cohen, Wheelwright, Skinner, *et al*., 2001). Our results suggest that even in the absence of marked social and communicative impairments signifying the potential need for a clinical diagnosis, those with higher levels of ASD traits may be more susceptible than others in the general population to socio-emotional and cognitive difficulties.

Overall, our findings are in line with past research linking ASD traits to social-cognitive difficulties and cognitive inflexibility, and provide unique insight into this relationship in typically developing adults. One limitation of the study is that the high and low ASD trait groups were not matched for age, gender, or IQ. While we cannot rule out potential confounding effects of IQ, statistical analysis revealed no significant confounding by gender or age. Thus, it is unlikely that our findings were adversely affected by confounding. A second limitation concerns the unbalanced presentation of negatively valanced stimuli. Due to the restricted range of basic positive emotions (e.g., happiness), the ERST presented a wider variety of negatively valenced facial expressions (e.g., fear, anger, disgust, and sadness). Previous investigations have reported profound emotion recognition deficits in individuals with ASDs, but only for basic negative emotions (Ashwin *et al*., 2006). Although somewhat speculative, it is possible that those with higher ASD traits experience similar difficulties in identifying negative expressions, and this may explain their poor performance on the ERST. Future research should seek to identify whether impaired rule-shifting is valence-specific for individuals with higher levels of ASD traits.

In conclusion, our findings show that there is considerable overlap between ASD trait constructs and various aspects of trait emotional intelligence. Moreover, our results suggest that flexible processing of emotional stimuli may be a critical feature of variation in ASD traits. Further examination of such processes in both clinical and sub-clinical ASD has the potential to further our understanding of the broader autism phenotype.

## Footnotes

1 Age and gender were included in all analyses and subsequently removed after returning non-significant results.

2 Squared semi-partial correlations for the four trait EI factors were Emotionality (*sr*² = .05), Sociability (*sr*² = .01), Well-being (*sr*² = .00), and Self-control (*sr*² = .00).

3 Most sub-facets were highly correlated, therefore, three facets were selected to provide the most parsimonious regression model (i.e., predictor variables with high correlations with the criterion and low inter-correlations). The full correlation matrix is available from the corresponding author.

4 Squared semi-partial correlations for the three trait EI facets were social awareness (*sr*² = .06), adaptability (*sr*² = .04), and empathy (*sr*² = .01).
